# Food Reservoir for *Escherichia coli* Causing Urinary Tract Infections

**DOI:** 10.3201/eid1601.091118

**Published:** 2010-01

**Authors:** Caroline Vincent, Patrick Boerlin, Danielle Daignault, Charles M. Dozois, Lucie Dutil, Chrissi Galanakis, Richard J. Reid-Smith, Pierre-Paul Tellier, Patricia A. Tellis, Kim Ziebell, Amee R. Manges

**Affiliations:** McGill University, Montréal, Québec, Canada (C. Vincent, C. Galanakis, P.-P. Tellier, P.A. Tellis, A.R. Manges); University of Guelph, Guelph, Ontario, Canada (P. Boerlin, R.J. Reid-Smith); Public Health Agency of Canada, Saint-Hyacinthe, Québec (D. Daignault, L. Dutil); INRS-Institut Armand-Frappier, Laval, Québec (C.M. Dozois); Public Health Agency of Canada, Guelph (R.J. Reid-Smith, K. Ziebell)

**Keywords:** Escherichia coli, molecular epidemiology, urinary tract infections, extraintestinal infections, antimicrobial resistance, retail meat, foodborne transmission, food reservoir, bacteria, research

## Abstract

Closely related strains of *Escherichia coli* have been shown to cause extraintestinal infections in unrelated persons. This study tests whether a food reservoir may exist for these *E. coli*. Isolates from 3 sources over the same time period (2005–2007) and geographic area were compared. The sources comprised prospectively collected *E. coli* isolates from women with urinary tract infection (UTI) (n = 353); retail meat (n = 417); and restaurant/ready-to-eat foods (n = 74). *E. coli* were evaluated for antimicrobial drug susceptibility and O:H serotype and compared by using 4 different genotyping methods. We identified 17 clonal groups that contained *E. coli* isolates (n = 72) from >1 source. *E. coli* from retail chicken (O25:H4-ST131 and O114:H4-ST117) and honeydew melon (O2:H7-ST95) were indistinguishable from or closely related to *E. coli* from human UTIs. This study provides strong support for the role of food reservoirs or foodborne transmission in the dissemination of *E. coli* causing common community-acquired UTIs.

Foodborne transmission of extraintestinal *E. coli* is common.

Extraintestinal infections caused by *Escherichia coli* cause serious illness and death. Every year, 6–8 million cases of uncomplicated urinary tract infections (UTI) occur in the United States and 130–175 million cases occur globally; >80% are associated with *E. coli* (*1**,**2*). The urinary tract is the most common source for *E. coli* causing bloodstream infections, which cause 40,000 deaths from sepsis each year in the United States (*1*). Uncomplicated UTIs alone are responsible for an estimated $1–$2 billion of direct healthcare costs in the United States annually (*1**,**2*). Antimicrobial drug resistance among extraintestinal *E. coli* is further adding to the cost of treating these infections (*3*). Drug-resistant infections often require more complicated treatment regimens and result in more treatment failures.

The immediate reservoir of *E. coli* that causes extraintestinal infections is the intestinal tract of the person. Although extraintestinal infections caused by *E. coli* are not usually associated with outbreaks, mounting evidence shows that extraintestinal *E. coli* may be responsible for community-wide epidemics. For instance, in 2001, we reported the discovery of *E. coli* O11/O77/O17/O73:K52:H18-ST69. This clonal group caused 11% of all *E. coli* UTIs and 49% of all trimethoprim/sulfamethoxazole-resistant *E. coli* UTIs in 1 California community over a 4-month period (*4*). It caused antimicrobial drug–resistant UTIs in Michigan, Minnesota, and Colorado (*5*), and pyelonephritis in several states (*6*). Other outbreaks of UTIs caused by *E. coli* have been described, including a large *E. coli* O15:K52:H1 outbreak in South London (*7*), clusters of cases in Copenhagen, Denmark, caused by *E. coli* O78:H10 (*8*), and cases in Calgary, Alberta, Canada, caused by an extended-spectrum β-lactamase-producing *E. coli* (*9*).

Identification of these outbreak strains has suggested that environmental sources, possibly contaminated meat and other foods, may play a role in the local spread of closely related *E. coli* strains. If there is a food animal reservoir for extraintestinal *E. coli*, then the use of antimicrobial agents in food animal production may select for antimicrobial drug–resistant forms of extraintestinal *E. coli* (*10**,**11*). Links between antimicrobial resistance and specific strains of extraintestinal *E. coli* in animal food products, specifically chicken meat, and human infections have been observed (*12**–**16*). In a previous study, we noted an increase in antimicrobial drug–resistant UTIs among women who report frequent chicken and pork consumption (*17*).

Evidence showing that food can be a reservoir for extraintestinal *E. coli* includes 1) community-based outbreaks of extraintestinal infections caused by epidemic strains of *E. coli* causing uncomplicated UTIs (*4**,**18*) and other severe infections (*6**,**19**,**20*); 2) the determination that these epidemic strains share antimicrobial drug susceptibility patterns and genotypes with isolates from retail meat (*12**–**15*); and 3) the epidemiologic association between retail meat consumption and intestinal acquisition of antimicrobial drug–resistant *E. coli* causing UTIs (*17*). On the basis of these observations, we hypothesize that retail chicken is the main reservoir for *E. coli* causing human extraintestinal infections.

## Methods

### Study Design

*E. coli* isolates from human clinical samples, restaurant/ready-to-eat foods, and retail meat were systematically sampled over the same period. Human clinical isolates and restaurant/ready-to-eat isolates were obtained from Montréal, Québec, Canada. Retail meat isolates from Québec and Ontario were included because women with infections were primarily from these regions. We hoped to maximize the probability that matching genotypes between *E. coli* from these 3 sources could be identified. *E. coli* isolates from each source were cultured and processed separately to prevent cross-contamination. The study protocol was approved by the McGill University Institutional Review Board (A01-M04-05A).

### Sampling of *E. coli* Causing Human UTIs

*E. coli* isolates from women with UTIs in Montréal from June 1, 2005, to May 30, 2007, were included. Women 18–45 years of age with a suspected UTI were enrolled. UTI was defined as the presence >2 relevant symptoms including dysuria, increased urinary frequency or urgency, pyuria, and hematuria and >10^2^ colony-forming units of *E. coli* per milliliter of clean-catch urine (*21*). A total of 1,395 consecutive UTI samples were obtained. Details about specimen culture and bacterial identification of *E. coli* are provided in Manges et al. (*18*). One *E. coli* isolate from each urine culture was arbitrarily selected for further analysis. If a woman had had recurrent UTIs, only the isolate from the first infection was included. The study sample (n = 353) of *E. coli* isolates was assembled in the following manner. All cephalothin-resistant *E. coli* (n = 19) were included. Isolates known to be members of a clonal group (n = 46) found to be closely related to or indistinguishable from other *E. coli* causing UTI in unrelated women were included (*4**,**18**,**22*) because we hypothesized that these *E. coli* would be more likely to be associated with food sources. A random sample of *E. coli* isolates resistant to >1 antimicrobial agents was assembled (n = 172). We chose to oversample resistant *E. coli*, as antimicrobial resistance has been associated with possible outbreaks of extraintestinal *E. coli* infections. A random sample of fully susceptible *E. coli* isolates (n = 116) was selected.

### Sampling of *E. coli* from Retail Meat

A total of 417 *E. coli* isolates from fresh, raw retail chicken, beef, and pork products were selected from the collection of the Canadian Integrated Program for Antimicrobial Resistance Surveillance (CIPARS), which monitors antimicrobial resistance in bacteria from meat obtained from grocery and other retail stores in several provinces in Canada (*23*). Isolates collected by the CIPARS in Montréal, areas of Québec outside Montréal, and parts of Ontario from January 1, 2005, to July 31, 2007, were included as follows. All CIPARS isolates from Montréal were included because all cases of UTI occurred in Montréal (n = 197). All CIPARS nalidixic acid–resistant *E. coli* from all regions of Canada were included (n = 24); these isolates have been associated with reduced susceptibility to fluoroquinolones. Randomly selected susceptible and resistant isolates from outside Montréal, including other regions of Québec and Ontario, were selected to better represent the possible sources of retail meat exposure for the UTI cases. The overall sampling fraction for retail chicken meat-associated isolates was ≈60%, given that our primary hypothesis focused on retail chicken meat. The sampling fraction for retail beef was 20% and for retail pork 20%. A strong association between extraintestinal *E. coli* clonal groups and antimicrobial resistance has been reported (*4**,**7**,**9**,**18*). Our targeted sampling fraction for antimicrobial resistance was 60% for each retail meat category; however, only 25% of retail beef isolates were resistant.

### Sampling of *E. coli* from Restaurant/Ready-to-Eat Food Sources

We included all 74 *E. coli* isolates from restaurant and ready-to-eat food sources for Montréal collected from January 1, 2005, to December 31, 2007, by the Division de l’Inspection des Aliments (*24**,**25*). These isolates were recovered from a range of prepared and ready-to-eat foods, including meat, fruit, vegetables, and other items. Isolates were collected as part of routine surveillance activities and from complaint-related inspections of restaurants and establishments offering ready-to-eat foods.

### Antimicrobial Drug Susceptibility

We determined the minimum inhibitory concentration values for 15 antimicrobial agents for all *E. coli* isolates by the broth microdilution method (*26*), using the Sensititre Automated Microbiology System (Trek Diagnostic Systems Ltd., Cleveland, OH, USA). National Antimicrobial Resistance Monitoring System (NARMS) susceptibility panel CMV1AGNF was used for *E. coli* testing. Human clinical and restaurant/ready-to-eat isolates were also evaluated for resistance to cephalothin and nitrofurantoin by a standard disk diffusion method (*27*). Isolates were defined as resistant, intermediate, or susceptible according to Clinical and Laboratory Standards Institute and NARMS guidelines (*23*). Isolates exhibiting intermediate resistance were interpreted as susceptible.

### Multilocus Variable Number Tandem Repeat Analysis

We performed multilocus variable number tandem repeat analysis (MLVA) on all isolates using capillary electrophoresis methods as described previously in Manges et al (*28*). Essentially, 8 loci were amplified in separate PCRs by using fluorescent primers. Raw fragment lengths for each locus were binned manually using a minimum threshold of ± 3 bp to distinguish alleles. *E. coli* CFT073, K12, and O157:H7 were used as positive controls. The set of 8 alleles for each isolate was defined as the MLVA profile.

### Enterobacterial Repetitive Intergenic Consensus Sequence 2 PCR Fingerprinting

*E. coli* isolates exhibiting indistinguishable MLVA profiles were compared by enterobacterial repetitive intergenic consensus sequence 2 PCR (ERIC2 PCR) fingerprinting (*29*). Isolates with fingerprints that were indistinguishable on visual inspection were grouped and selected for further typing.

### Clonal Group Definition

A clonal group was defined as >2 *E. coli* isolates exhibiting indistinguishable MLVA and ERIC2 PCR patterns. We focused only on groups identified by MLVA and ERIC2 PCR that contained members from >1 source. Groups containing isolates from retail meat and restaurant/ready-to-eat food sources were included to determine whether related extraintestinal *E. coli* from retail meat isolates could be identified in prepared food. These groups were given a designation that included the serogroup and multilocus sequence type (MLST), as in serogroup O25:H4 and ST131 (O25:H4-ST131). Selected isolates representing each clonal group were chosen and evaluated by pulsed-field gel electrophoresis (PFGE), serotyping, MLST, and phylogenetic typing to confirm the identities of these clonal groups and to define their within-group variability.

### Pulsed-Field Gel Electrophoresis

The standard Centers for Disease Control and Prevention protocol for molecular subtyping of *E. coli* O157:H7 by PFGE was used (*30*). PFGE of *Xba*I- and *Not*I-digested DNA was performed on selected isolates belonging to each clonal group. Isolates exhibiting identical PFGE patterns were considered genetically indistinguishable, those exhibiting 1–3 band differences were considered closely related, and those exhibiting 4–6 band differences were considered possibly related (*31*).

### Serotyping

The Public Health Agency of Canada Laboratory for Foodborne Zoonoses performed O- and H-serotyping using established protocols. Isolates that did not react with O antiserum were classified as nontypeable (ONT), and those that were nonmotile were denoted NM.

### MLST and Phylotyping

MLST on selected *E. coli* isolates was performed as previously described (*32*). Gene amplification and sequencing were performed by using the primers specified at the *E. coli* MLST website (http://mlst.ucc.ie/mlst/dbs/Ecoli). Allelic profile and sequence type determinations were assigned according to this website’s scheme. Determination of the major *E. coli* phylogenetic groups (A, B1, B2, and D) was performed by multiplex PCR (*33*).

### Statistical Analyses

Proportions and 95% confidence intervals for proportions were estimated. Differences in proportions were assessed by χ^2^ tests; statistical significance was defined as a p value <0.05. All analyses were conducted using Stata version 9.0 (StataCorp LP, College Station, TX, USA).

## Results

### Final Sample Assembly

We analyzed 844 *E. coli* isolates obtained from human UTIs (n = 353), retail meat (n = 417), and restaurant/ready-to-eat foods (n = 74). Table 1 contains details regarding the year of isolation, geographic location, and specific meat or food source.

**Table 1:** Sources of 844 *Escherichia coli* isolates collected and analyzed in Canada, by year and location, 2005–2007*

Source	Total no. (%) isolates	Year, no. (%) isolates				Location, no. (%) isolates		
		2005	2006	2007		Quebec	Ontario	Other†
Clinical								
UTI	353 (42)	103 (29)	175 (50)	75 (21)		353 (100)	0	0
Retail meat								
All	417 (49)	178 (43)	158 (38)	81(19)		264 (63)	139 (33)	14 (3)
Chicken	253 (61)	107 (42)	101 (40)	45 (18)		141 (56)	99 (39)	13 (5)
Beef	82 (20)	37 (45)	26 (32)	19 (23)		81 (99)	1 (1)	0
Pork	82 (20)	34 (41)	31 (38)	17 (21)		42 (51)	39 (48)	1 (1)
Restaurant/ready-to-eat foods								
All	74 (9)	19 (26)	33 (45)	22 (30)		74 (100)	0	0
Chicken	21 (28)	7 (33)	6 (29)	8 (38)		21 (100)	0	0
Beef	13 (18)	3 (23)	6 (46)	4 (31)		13 (100)	0	0
Pork	5 (7)	0	4 (80)	1 (20)		5 (100)	0	0
Fish/seafood	6 (8)	2 (33)	2 (33)	2 (33)		6 (100)	0	0
Other meat‡	9 (12)	1 (11)	7 (78)	1 (11)		9 (100)	0	0
Other food§	20 (27)	6 (30)	8 (40)	6 (30)		20 (100)	0	0
Total	844 (100)	300 (36)	366 (43)	178 (21)		691 (82)	139 (16)	14 (2)

*UTI, urinary tract infection. †British Columbia (n = 4) and Saskatchewan (n = 10). ‡Bison, lamb, duck, and snail. §Fruits (honeydew melon), vegetables, cheese, rice, couscous, and pasta.

### Clonal Group Identification and Characterization

Seventeen clonal groups were identified (containing a total of 72 isolates). Eleven groups contained isolates from human infections and retail meat sources; 5 groups contained isolates from retail meat and restaurant/ready-to-eat food sources; and 1 group contained isolates from restaurant/ready-to-eat food and human infections. Fifty-seven representative isolates were selected for evaluation by PFGE, MLST, serotyping, and phylotyping (Table 2).

**Table 2:** Characteristics of *Escherichia coli* clonal groups identified within isolates from 3 types of samples, Canada, 2005–2007*†

Group and strain	Type of sample	Isolate source	Location‡	Year	Genotype				MLST ST	Serotype	
					MLVA	ERIC2	*Xba*I PFGE				
1											
EC01DT06-1737-01	Retail meat	Chicken	Montreal	2006	1.033	33.01	33A.0		131	O25:H4	
MSHS 161	Clinical	Human	Montreal	2005	1.033	33.01	33A.0		131	O25:H4	
MSHS 1134A	Clinical	Human	Montreal	2007	1.033	33.01	33A.1		131	O25:H4	
2											
68616.01	RTE	Honeydew	Montreal	2005	1.018	18.01	18A.0		95	O2:H7	
MSHS 100	Clinical	Human	Montreal	2005	1.018	18.01	18A.0		95	O2:H7	
MSHS 186	Clinical	Human	Montreal	2005	1.018	18.01	18A.0		95	O2:H7	
MSHS 811	Clinical	Human	Montreal	2006	1.018	18.01	18A.0		95	O2:H7	
MSHS 1229	Clinical	Human	Montreal	2007	1.018	18.01	18A.1		95	O2:H7	
MSHS 95	Clinical	Human	Montreal	2005	1.018	18.01	18A.2		95	O2:H7	
MSHS 1062	Clinical	Human	Montreal	2007	1.018	18.01	18A.2		95	O2:NM	
MSHS 782	Clinical	Human	Montreal	2006	1.018	18.01	18A.4		95	O2:H7	
MSHS 819	Clinical	Human	Montreal	2006	1.018	18.01	18A.4		95	O2:H7	
3											
EC01DT05-0789-01	Retail meat	Chicken	Ontario	2005	1.023	23.01	23A.0		117	O114:H4	
MSHS 1014A	Clinical	Human	Montreal	2007	1.023	23.01	23A.5		117	O114:H4	
EC01DT05-0224-01	Retail meat	Chicken	Ontario	2005	1.023	23.01	23B		117	ONT:NM	
EC01DT06-1887-01	Retail meat	Chicken	Montreal	2006	1.023	23.01	23C		117	O143:H4	
EC01DT07-0956-01	Retail meat	Chicken	Other	2007	1.023	23.01	23D		117	O53:H4	
EC01DT05-1700-01	Retail meat	Chicken	Quebec	2005	1.023	23.01	NT		117	O160:H4	
EC01DT07-1050-01	Retail meat	Chicken	Ontario	2007	1.023	23.01	NT		117	O45:H4	
EC01DT07-1090-01	Retail meat	Chicken	Montreal	2007	1.023	23.01	NT		117	O24:H4	
MSHS 133	Clinical	Human	Montreal	2005	1.023	23.01	NT		117	O24:NM	
4											
EC01DT06-0649-01	Retail meat	Pork	Montreal	2006	1.116	116.01	116A		69	O17/73/106:H18	
MSHS 719	Clinical	Human	Montreal	2006	1.116	116.01	116C		69	O44:H18	
MSHS 956	Clinical	Human	Montreal	2007	1.116	116.01	116D		69	ONT:H18	
5											
EC01DT05-1012-01	Retail meat	Pork	Ontario	2005	1.102	102.01	102A		493	O4:H5	
MSHS 769	Clinical	Human	Montreal	2006	1.102	102.01	102B		493	O4:H5	
6											
EC01DT06-1265-01	Retail meat	Beef	Montreal	2006	2.107	107.01	107A		401	O36:NM	
76083.08	RTE	Chicken	Montreal	2007	2.107	107.01	107B		401	O36:NM	
7											
EC01DT06-0274-01	Retail meat	Chicken	Quebec	2006	2.097	97.01	97A		295	O172:H16	
79287	RTE	Chicken	Montreal	2007	2.097	97.01	97B		295	O172:H16	

*MLST, multilocus sequence typing; MLVA, multilocus variable number tandem repeat analysis; ERIC2, enterobacterial repetitive intergenic consensus sequence 2; PFGE, pulsed-field gel electrophoresis; ST, sequence type; RTE, restaurant/ready-to-eat foods; NT, nontypeable; ONT, serogroup nontypeable; NM, non-motile; UNK, unknown. An expanded version of Table 2 containing all isolates is available online at www.cdc.gov/EID/content/16/1/zzz-T2.htm. †All isolates in groups 1, 2, and 5 were phylotype B2; all isolates in groups 3, 4, and 8 were phylotype D; all isolates in groups 6, 9, 10, and 11, as well as isolate MSHS 689 in group 17, were phylotype A; all isolates in groups 7, 12, 13, 14, 15, and 16, as well as isolate EC01DT05-0469-01 from group 17, were phylotype B1. ‡Other locations were Saskatchewan or British Columbia.

**Figure 1:**
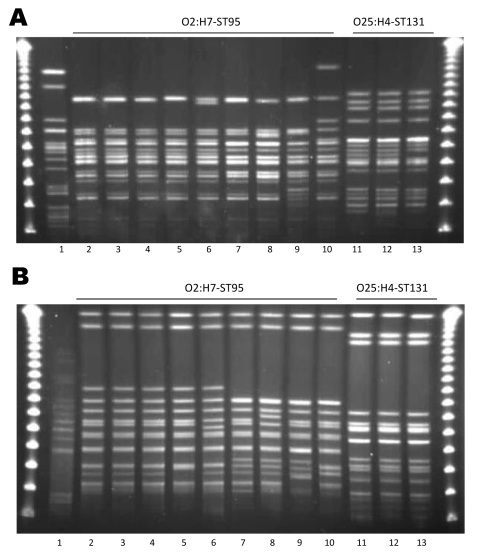
Pulsed-field gel electrophoresis patterns for *Escherichia coli* O2:H7-ST95 and *E. coli* O25:H4-ST131. A) *Xba*I; B) *Not*I. Lane 1 is the positive control *E. coli* O11:H18-ST69 (SEQ102); lane 2 is an *E. coli* O2:H7-ST95 isolate from a restaurant sample of honeydew melon (68616.01); lanes 3–10 are isolates from human urinary tract infection cases (UTIs; lane 3, MSHS 100; lane 4, MSHS 186; lane 5, MSHS 811; lane 6, MSHS 1229; lane 7, MSHS 95; lane 8, MSHS 1062; lane 9, MSHS 782; lane 10, MSHS 819); lane 11 is an *E. coli* O25:H4-ST131 isolate from a retail chicken sample (EC01DT06-1737-01); and lanes 12 and 13 are *E. coli* isolates from human UTIs (lane 12, MSHS 161; lane 13, MSHS 1134A). Outer lanes are pulsed-field molecular weight markers.

On the basis of PFGE patterns, we identified 2 clonal groups (group 1 and group 2) that contained genetically indistinguishable isolates and 1 clonal group (group 3) that contained closely related isolates from food sources and human UTIs. Group 1 contained *E. coli* characterized as O25:H4-ST131, which was identified in 1 sample of retail chicken meat and in 2 cases of human infection. The *Xba*I PFGE patterns of the human isolate (MSHS 161) and the retail chicken isolate (EC01DT06-1737-01) were indistinguishable, and the second human isolate (MSHS 1134A) differed by 1 band from the other 2 patterns (Figure 1, panel A). The *Not*I PFGE patterns of the 2 human isolates, which were indistinguishable, differed from the retail chicken isolate by a single band (Figure 1, panel B). The retail meat isolate from this group was susceptible to all antimicrobial agents tested, while 1 of the 2 isolates from human infections was resistant to cephalothin and the second was resistant to ampicillin, streptomycin, sulfisoxazole, and tetracycline.

Group 2 contained *E. coli* characterized as O2:H7-ST95; one isolate was from a restaurant/ready-to-eat food source (a honeydew melon) and 8 isolates were from cases of human infection. The *Xba*I PFGE patterns were indistinguishable for 3 of the human infection isolates (MSHS 100, 186, and 811) and the restaurant/ready-to-eat food isolate (68616.01); the other 5 O2:H7-ST95 isolates differed by 1 band (MSHS 1229), two bands (MSHS 95 and MSHS 1062), and 4 bands (MSHS 782 and MSHS 819) from the food source isolate, respectively (Figure 1, panel A ). The *Not*I PFGE patterns for MSHS 100 and MSHS 186 were indistinguishable from the restaurant/ready-to-eat isolate, and the other human infection isolates differed by 1 to 7 bands (Figure 1, panel B). The *E. coli* isolate from the food source was fully susceptible, as were most isolates from the human infections, except for 2 (one was resistant to ampicillin, and the second to ampicillin, sulfisoxazole, and trimethoprim/sulfamethoxazole).

**Figure 2:**
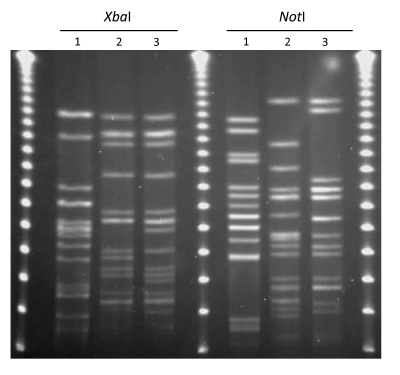
*Xba*I and *Not*I pulsed-field gel electrophoresis patterns for *Escherichia coli* O114:H4-ST117 (lanes 2 and 3). Lane 1 is the positive control *E. coli* O11:H18-ST69 (SEQ102), lane 2 is an *E. coli* O25:H4-ST131 isolate from a retail chicken sample (EC01DT06-1737-01), and lane 3 is an *E. coli* isolate from a human urinary tract infection case (MSHS 1014A). Outer and center lanes are pulsed-field molecular weight markers.

Group 3 contained *E. coli* characterized as O114:H4-ST117; one isolate was from retail chicken meat and the second was from a human UTI. The *Xba*I PFGE patterns of the human infection isolate (MSHS 1014A) and retail meat isolate (EC01DT05-0789-01) differed by 5 bands (Figure 2). The *Not*I PFGE patterns differed by >6 bands (Figure 2). Both isolates were fully susceptible. In addition to shared PFGE patterns, these 3 groups of *E. coli* shared the same MLSTs, serotypes, and phylotypes.

The clonal group characterized as *E. coli* O17/O73/O77:H18-ST69, also known as clonal group A (*4*), was identified in human and retail meat samples, although closely related PFGE patterns were not observed (group 4, Table 2). Three other groups (groups 5–7, Table 2), characterized as *E. coli* O4:H5-ST493, O36:NM-ST401, and O172:H16-ST295, exhibited shared MLSTs, serotypes, and phylotypes, but the PFGE patterns were not related.

## Discussion

We report the identification of *E. coli* isolates from retail chicken and other food sources that are indistinguishable from or closely related to isolates from human UTIs. Our a priori hypothesis, based on results from previous studies, suggests that retail meat, specifically retail chicken meat, could be a reservoir for *E. coli* causing human extraintestinal infections. This study provides strong support for this hypothesis on the basis of genetic similarities between food and human clinical isolates.

Johnson et al. have proposed that antimicrobial drug–resistant *E. coli* from human feces (and human bloodstream infections) tend to be more similar to antimicrobial-resistant and -susceptible *E. coli* from retail poultry meat sources (*14**,**15*). These observations indicate that the selection of resistant *E. coli* is more likely to occur in the animal food reservoir than in humans. In this study, we observed that genetically related *E. coli* from food sources and human infections tended to be susceptible, suggesting that both resistant and susceptible isolates causing UTIs in women may be transmitted through the food supply. Our study also identified members of the O2:H7-ST95 group, previously associated with extraintestinal disease in both humans and avian hosts (*34*). The O2:H7-ST95 food source isolate from this study was from a honeydew melon. Potential origins of this *E. coli* contamination could include human or food animal sources.

The *E. coli* O25:H4-ST131 clonal group, also identified in this study, has been associated with extended spectrum β-lactamase production and fluoroquinolone resistance and has been found across Europe and in Canada (*18**,**35**–**37*). The *E. coli* O25:H4-ST131 isolates identified in this study are susceptible; however, because this clonal group may be found in a food animal reservoir and transmitted by food, amplification and transmission of these highly resistant organisms could be possible. Extended spectrum β-lactamase-producing *E. coli* have not yet been identified by CIPARS (*23**,**38**,**39*).

This study was ecologic in design and presents several limitations. Epidemiologic information on the UTI cases was not available. Information on travel, history of antimicrobial drug use, dietary information, and other factors would have been useful to describe the study population and to assess the significance of other possible transmission routes that might explain our results. The study also oversampled retail chicken meat and consequently undersampled isolates from retail pork and beef. It is possible that closely related clonal groups could be identified that contain isolates from both human infections and pork or beef samples. Because of insufficient power in our sampling strategy we could not exclude the existence of these groups; additional sampling of isolates from retail pork and beef are underway to address this question. Despite oversampling isolates from retail chicken meat, we observed that 82% (a greater fraction than the 60% sampling fraction) of *E. coli* belonging to the 17 clonal groups were associated with retail chicken meat. We also oversampled antimicrobial drug-resistant isolates; however, most (53%) isolates that belonged to a clonal group were fully susceptible. Even though the size and scope of this study was limited, we were able to detect several instances of groups containing closely related isolates from human and food sources. It is therefore probable that a food reservoir exists and that foodborne transmission of extraintestinal *E. coli* is common.

The identification of 2 clonal groups containing isolates from retail chicken meat and human infections supports our a priori hypothesis. We cannot exclude the possibility that food source isolates were present because of human contamination during food production, processing or handling, even though it is very unlikely. Subsequent research will help determine whether these *E. coli* occur in a food animal reservoir or whether transfer of these *E. coli* results from contamination during food processing or preparation and reflects human-to-human transmission by food.

This study demonstrates that some *E. coli* from retail chicken meat and other food sources are closely related to *E. coli* causing human UTIs. Since a food animal reservoir apparently exists for *E. coli* that cause urinary tract and other extraintestinal infections, this further reinforces the need for responsible antimicrobial drug stewardship in veterinary medicine and food animal production as well as in human medicine.
